# Comparative Analysis of Volatile Components Changes in Horse Fat During Dry Fractionation by E-Nose, HS/GC-IMS and SPME/GC-MS

**DOI:** 10.3390/foods15172959

**Published:** 2026-08-23

**Authors:** Daihao Huang, Mingfang Hua, Gulnur Nurymkhan, Samat Kassymov, Almagul Nurgazezova, Yernaz Yermekov, Maral Iskakova, Botakoz Kulushtayeva, Laila Bakirova, Nazerke Muslimova, Aigul Maizhanova, Yuan Gao, Xiuzhu Yu

**Affiliations:** 1 Engineering Research Center of Grain and Oil Functionalized Processing, College of Food Science and Engineering, Northwest A&F University, 22 Xinong Road, Yangling District, Xianyang 712100, China; aaahdh@nwafu.edu.cn (D.H.); 13625667731@163.com (M.H.); xiuzhuyu@nwafu.edu.cn (X.Y.); 2 Research Department of Food Technology, Shakarim University, Glinka Street, 20A, Semey 071412, Kazakhstan; gulnu-n@mail.ru (G.N.); almanya1975@mail.ru (A.N.); m.iskakova@shakarim.kz (M.I.); kulushtaeva_89@mail.ru (B.K.); bakirova2010@mail.ru (L.B.); muslimova.n.r@mail.ru (N.M.); fquekm2710@mail.ru (A.M.); 3 Department of Food Security, Kozybayev University, 114 Zhumabayev Street, Petropavlovsk 150000, Kazakhstan; yernazyermekov@outlook.com; 4 Department of Agricultural Product Processing, BEENAR LABS LLP, Astana 010000, Kazakhstan

**Keywords:** horse fat, dry fractionation, volatile compounds, E-nose, HS/GC-IMS, SPME/GC-MS

## Abstract

Horse fat is a valuable lipid source for traditional meat products, but its flavor quality is remains unclear during processing conditions. To investigate the effect of dry fractionation on the volatile profile of refined horse fat, samples obtained at different temperature were analyzed using an E-nose, HS/GC-IMS, and SPME/GC-MS. The E-nose results showed that the volatile profiles of the samples differed markedly, with the solid fractions obtained at 25 and 15 °C (S25 and S15) exhibiting stronger sensor responses and clustering together, whereas the liquid fraction at 5 °C (L5) showed the weakest overall signals. HS/GC-IMS identified total 55 volatile compounds, mainly aldehydes, alkenes, esters, alcohols, ketones, and nitrogen-containing compounds. Compared with refined horse fat, S25 and S15 were enriched in ketones, sulfur-containing compounds, and nitrogen-containing compounds, while L5 was dominated by alcohols and aldehydes. SPME/GC-MS identified 53 volatile compounds, with aldehydes as the main class in all samples. S5 showed the highest number of volatile species and the richest aroma profile, whereas S25 and S15 showed the lowest complexity. The total volatile content ranked as refined horse fat > S5 > L5 > S15 > S25, and ester content followed the order S5 > L5 > refined horse fat > S25 > S15. Overall, dry fractionation temperature significantly regulated the distribution of volatile compounds in horse fat. These findings may provide a theoretical basis for the potential application of horse fat in food processing and product development.

## 1. Introduction

Kazakhstan has historically relied on horse husbandry as an important part of its nomadic economy, and horse meat is the country’s third largest meat product, with an annual output of approximately 126,000 tons [1]. As a by-product of horse processing, horse fat has attracted increasing attention because it contains bioactive components and nutritional lipids [2]. It is obtained from horse adipose tissue and has been used in several Asian countries for its anti-inflammatory, antibacterial, and antipruritic properties [3,4]. It is also rich in unsaturated fatty acids, especially linoleic acid and α-linolenic acid [5,6]. Despite these potential values, the utilization of Kazakh horse fat remains limited. Most of it is discarded with slaughter by-products or used only for low-value feed and industrial purposes. Its application in the food and cosmetic industries is still underdeveloped [1,7]. More importantly, its sensory flavor characteristics may hinder its further development and wider utilization [8,9].

Dry fractionation is an environmentally friendly and simple method that separates lipids based on melting point differences without using solvents or additional reagents [10,11]. Recent studies have demonstrated that dry fractionation can be used to precisely tailor the physicochemical properties and volatile profiles of lipid systems for food applications [10,12]. For example, by optimizing dry fractionation conditions at 44 °C for 8 h with a cooling rate of 6 °C/h and combining the process with micro-oxidation at 150 °C for 40 min under 130 L/h airflow, a functional beef tallow with lower saturation, improved flavor, and better storage stability was successfully obtained. This study showed that fractionation could promote the enrichment of unsaturated fatty acids and reshape volatile compound distribution, thereby enhancing the sensory quality of tallow [11]. In addition, desirable aroma is a key indicator in edible oil quality evaluation [13]. It reflects the overall quality of horse fat and strongly affects consumer sensory experience, market acceptance, and competitiveness.

Electronic nose (E-nose), headspace gas chromatography-ion mobility spectrometry (HS/GC-IMS), and headspace solid-phase microextraction coupled with gas chromatography-mass spectrometry (SPME/GC-MS) have been increasingly employed for the analysis of volatile compounds in food samples [14,15]. E-nose is a biomimetic sensing tool that consists of a sensor array and a pattern recognition system. It converts gas-induced changes into measurable electrical signals through sensors with overlapping selectivity [16,17]. HS/GC-IMS enables rapid analysis, efficient separation, and direct visualization, which makes it suitable for comparing volatile profiles among samples [18,19]. SPME/GC-MS can identify and quantify volatile compounds with high sensitivity and reliable structural information, and is therefore widely used in flavor analysis [20,21]. Together, these three techniques provide complementary information for a more comprehensive characterization of volatile compounds in food.

Recent studies have demonstrated the value of combining these methods to resolve aroma differences across food systems. For instance, in Huangshui-related samples, E-nose, HS-GC-IMS, and HS-SPME-GC-MS were used together to compare Huangshui, fermented grains, pit mud, and Nongxiangxing baijiu, and 35 volatile organic compounds were identified as key discriminatory markers [22]. In brined smoked goat meat, E-nose and HS-SPME-GC-MS successfully distinguished the effects of apple, oak, and mesquite wood chips, revealing higher levels of smoke-derived phenolics such as guaiacol, phenol, o-cresol, and 2-methoxy-5-methylphenol in the oak- and mesquite-smoked groups [23]. In sea urchin gonads, integrated E-nose and HS-SPME-GC-MS analysis uncovered clear sex-dependent odor fingerprints and distinct enrichment patterns of volatile compounds, including hydrocarbons, terpenoids, aldehydes, ketones, and esters [24]. These examples indicate that combined instrumental approaches can effectively capture both overall aroma patterns and specific marker compounds in food products.

However, the changes in flavor profiles of refined horse fat produced by dry fractionation at different temperatures remain unclear. In particular, the combined application of E-nose, HS-GC-IMS, and HS-SPME-GC-MS has not been systematically investigated. This integrated approach may provide a more comprehensive characterization of the chemical changes occurring during fractionation and improve our understanding of the dynamic changes in volatile compounds.

To address this gap, the present study first used E-nose to distinguish the flavor differences among refined horse fat fractions. Then, HS-GC-IMS and HS-SPME-GC-MS were applied to identify the major volatile compounds, determine their relative contents, and compare the changes in volatile profiles among fractions. The findings may improve our understanding of the aroma characteristics and odor sources of refined horse fat and may also support the development of horse fat.

## 2. Materials and Methods

### 2.1. Materials

The refined horse fat samples used in this study were kindly provided by Shakarim University, Kazakhstan. 2-octanol standard were purchased from Shanghai Macklin Biochemical Technology Co., Ltd. (Shanghai, China).

### 2.2. Dry Fractionation Process for Refined Horse Fat Used for Traditional Kazakhstan Sausages

Refined horse fat was fractionated according to the method described by previous research with minor modification [11]. Briefly, 200 g of refined horse fat was placed in a beaker, which was then set in a water bath equipped with magnetic stirring. The fat was heated at 60 °C with continuous stirring for 1 h to eliminate its crystal memory. The water bath temperature was then adjusted to 25 °C, and the sample was held at this temperature for 9 h to allow complete crystallization. After crystallization, the mixture was centrifuged at 12,840 *g* for 10 min to separate the solid and liquid phases, and the supernatant was collected as the liquid fraction (L25), while the precipitate was collected as the solid fraction (S25). The same procedure was repeated at crystallization temperatures of 15 °C and 5 °C to obtain the corresponding liquid fractions (L15 and L5) and solid fractions (S15 and S5). All separated horse fat fractions were stored at 4 °C for 48 h before being analyzed.

Notably, the separation rate, physicochemical indices, and lipid composition of the refined horse fat fractions were investigated in our preliminary research. All quality parameters met the national standards. Therefore, the present study focused only on volatile compounds.

### 2.3. Electronic Nose (E-Nose) Analysis of Different Refined Horse Fat Fractions

For electronic nose (E-nose) analysis, a 2 mL horse oil was placed in a 40 mL headspace vial. A PEN3 electronic nose system (AIRSENSE Analytics, Schwerin, Germany), equipped with an array of 10 metal oxide semiconductor (MOS) sensors, was employed for analysis. The sampling needle was inserted to approximately two-thirds of the vial height. The headspace gas was then introduced into the sensor chamber at flow rate of 200 mL/min, sensor cleaning time of 300 s, the sensor responses were recorded for a detection time of 60 s. Data collection was performed after the electronic nose response curves had stabilized, and the resulting data were used for analysis.

### 2.4. HS/GC–IMS Analysis of Different Refined Horse Fat Fractions

Analysis of the samples was performed using a gas chromatography–ion mobility spectrometry (GC-IMS) system (FlavourSpec^®^, G.A.S., Dortmund, Germany). Both the carrier gas and the drift gas were high-purity nitrogen (≥99.999%). A 3 mL refined horse fat fraction sample was placed in a 20 mL headspace vial. The vial was incubated at 85.0 °C with stirring at 500 r/min for 20.0 min. The injection needle was heated to 90.0 °C to prevent sample condensation at the tip. The injection flow rate was 51.0 mL/min. The needle penetration depth into the vial was set to 15.0 mm. The pre-injection purge time was 5.0 s. The pre-injection dwell time was 0.5 s. The post-injection purge time was 10.0 s.

The flow rate program was set as follows: The initial flow rate was 2.0 mL/min and held for 2 min. It was then linearly increased to 10.0 mL/min within 10 min. After that, the flow rate was further increased to 100.0 mL/min. At 20 min, it was raised to 150.0 mL/min and maintained until the end of the analysis. The drift gas flow rate was kept constant at 75.0 mL/min. The separation time for a single run was 30 min.

Volatile organic compounds were identified by comparing their drift time (DT) and retention index (RI) with data of standard compounds in the GC-IMS database and the NIST 2020 library.

### 2.5. SPME/GC-MS Analysis of Different Refined Horse Fat Fractions

A 2.50 g portion of refined horse fat fraction was accurately weighed into a 20 mL headspace vial, and 20 μL of an internal standard solution (0.54 mg/mL 2-octanol) was added. A preconditioned 50/30 μm DVB/CAR/PDMS SPME fiber (Supelco, Bellefonte, PA, USA) was used. The extraction temperature was 83 °C, the equilibration time was 40 min, the extraction time was 59 min, and the desorption time was 5 min.

GC conditions: Inlet temperature: 250 °C; carrier gas: He, flow rate 1.0 mL/min, splitless mode; column: HP-5MS (30 m × 0.25 mm, 0.25 μm). Oven temperature program: initial temperature 35 °C (hold 3 min), ramp to 60 °C at 4 °C/min (hold 5 min), ramp to 90 °C at 4 °C/min (hold 2 min), ramp to 135 °C at 6 °C/min (hold 7 min), then ramp to 300 °C at 6 °C/min.

MS conditions: Ion source: EI; ion source temperature: 230 °C; electron energy: 70 eV; quadrupole temperature: 150 °C; mass scan range (*m*/*z*): 30–500 *m*/*z*; mass spectral library: NIST11; scan mode: full scan.

### 2.6. Statistical Analysis

At least three to five independent tests were performed, and the results are presented as the mean ± standard deviation (SD). One-way analysis of variance (ANOVA) was conducted on the different refined horse fat fraction samples using SPSS 27.0.1. Differences between groups were considered statistically significant at *p* < 0.05. E-nose data were analyzed using specialized software. Partial least squares discriminant analysis (PLS-DA) and VIP plots were generated using MetaboAnalyst 6.0. All other graphs were prepared using Origin 2024b (OriginLab Corporation, Northampton, MA, USA).

## 3. Results

### 3.1. E-Nose Analysis

**Figure 1:**
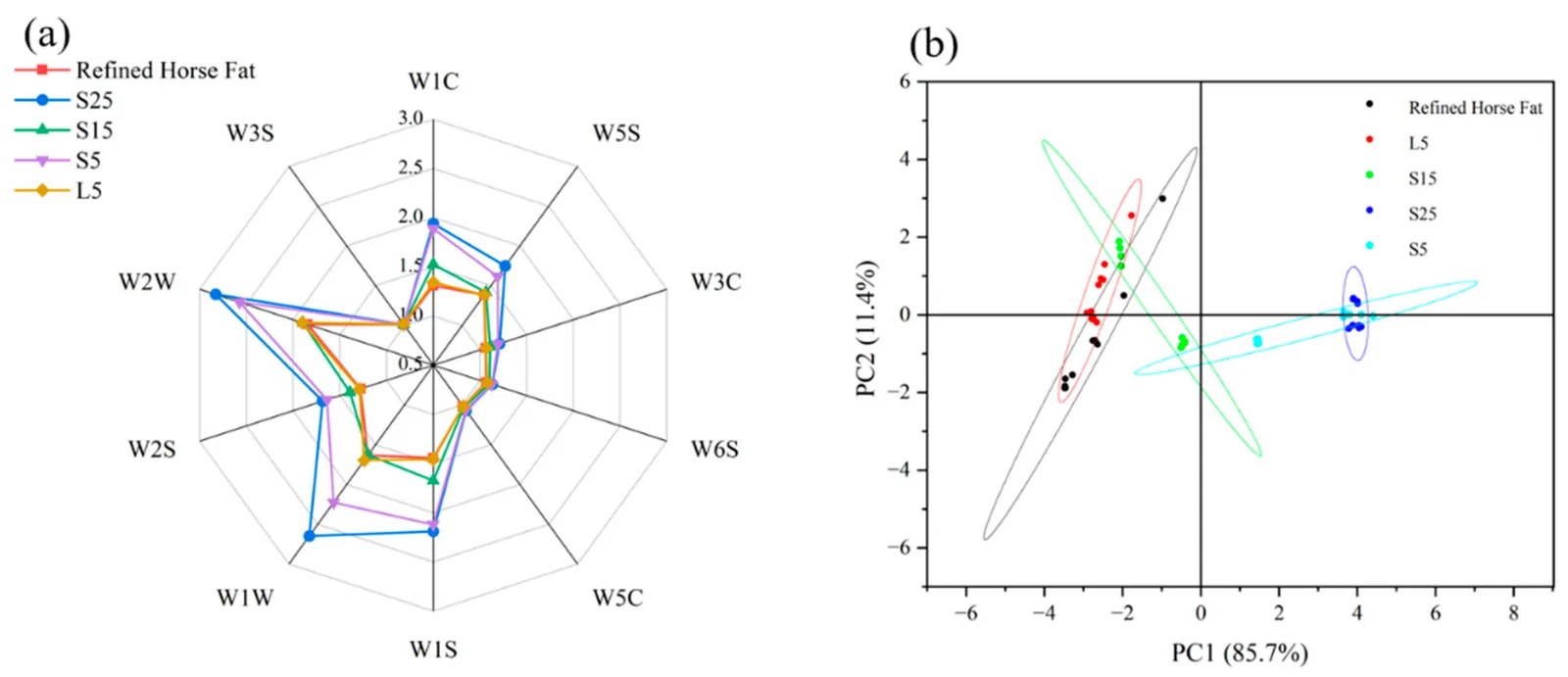
Radar chart (**a**) and principal component analysis (**b**) of E-nose data for different horse fat fractions.

The E-nose was employed to rapidly capture the overall volatile odor profile of the samples. In general, each sensor shows differential sensitivity toward specific classes of volatile compounds, and the resistance ratio was used as the response signal [25]. To further explore the effect of dry fractionation temperature on the volatile flavor characteristics of refined horse fat fractions, E-nose analysis was conducted to compare the aroma profiles of samples obtained at different temperature points. As shown in Figure 1, the volatile profiles of raw horse fat and the fractions obtained at different temperatures differed markedly.

The response patterns of S25, S15, S5, and L5 were clearly separated across sensors, indicating that fractionation temperature and phase state strongly influenced volatile compound distribution.

Among the sensors, W1C, W5S, W1W, and W2W contributed most to sample discrimination [20]. W1C, which is sensitive to aromatic compounds and some esters, showed higher responses for S25 and S15, suggesting greater enrichment of aroma-related volatiles in the solid fractions obtained at higher temperatures. S25 and S15 showed stronger responses on these sensors, indicating higher levels of aroma-related, sulfur-containing, roasted, and fruity volatiles [16]. W5S associated with nitrogen oxides and irritating volatiles, gave the highest response in S25, followed by S15, suggesting that higher-temperature solid fractions contained more nitrogen oxide- or irritation-related volatiles [18]. W1W and W2W which are related to sulfur-containing compounds and certain alcohols, ketones, and esters, also showed more strongly to S25 and S15, implying better retention of meaty, onion-like, roasted, and fruity odor-active compounds in these fractions [26]. In contrast, S5 showed weaker responses, while L5 exhibited the lowest signals across most sensors, indicating a reduced abundance of volatile flavor compounds in the liquid fraction. The different responses of S5 and L5 further confirmed the redistribution of volatiles in refined horse fat fractions between the solid and liquid phases during fractionation.

Principal component analysis (PCA) further supported these results. The samples were clearly separated in the score plot, with S25 and S15 clustered in one region and S5 and L5 separated on the opposite side, demonstrating that fractionation temperature and phase state collectively influenced the volatile flavor profile of horse fat. Overall, decreasing fractionation temperature weakened the signals associated with aroma, sulfurous, and roasted notes in the solid fractions, while increasing the flavor differences between the solid and liquid phases [27,28]. These findings indicate that dry fractionation can effectively modulate the migration and enrichment of volatile compounds, thereby helping optimize the flavor characteristics of horse fat.

### 3.2. Identification of Volatile Compounds by HS/GC-IMS

**Figure 2:**
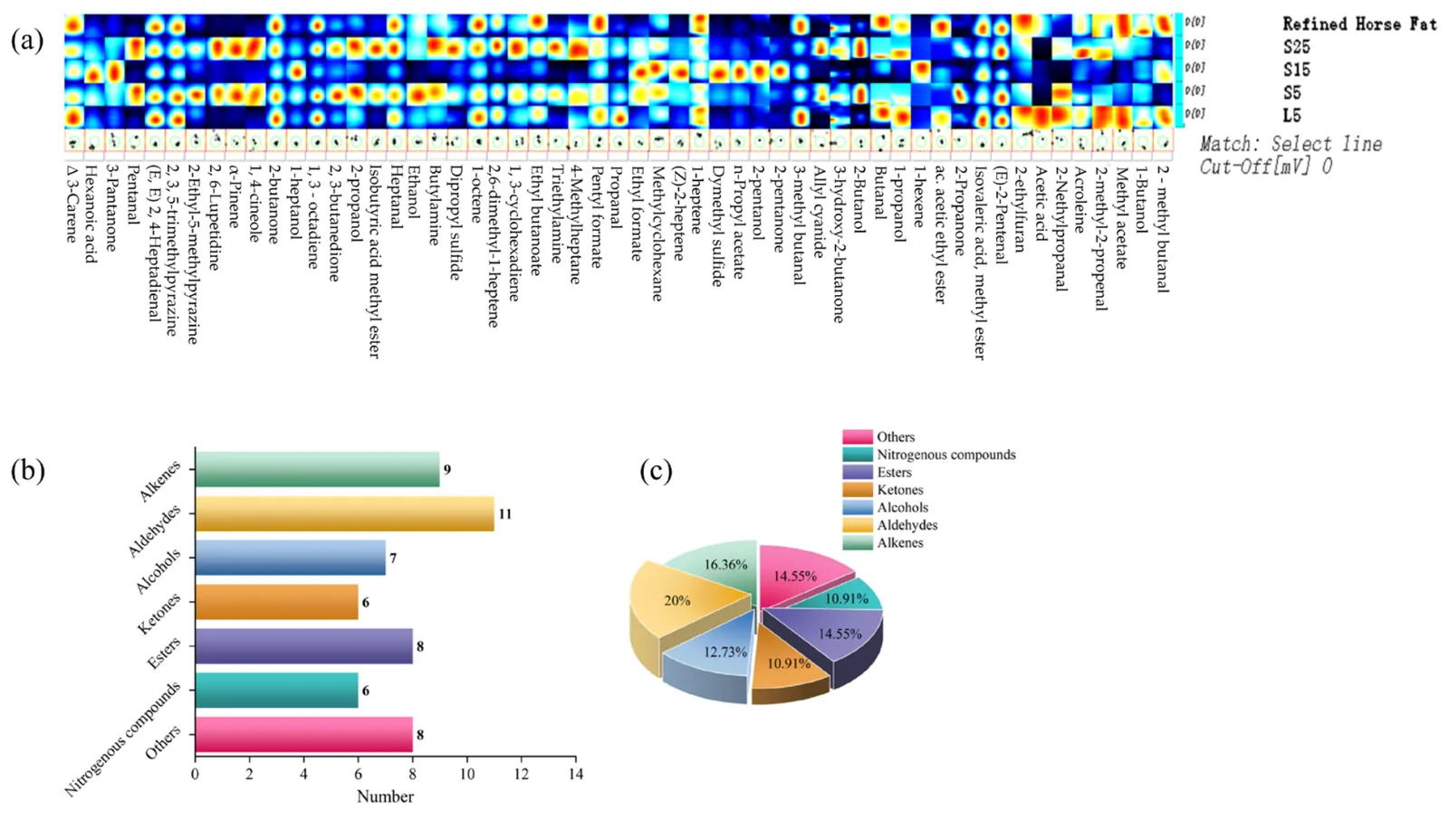
The VOCs of different horse fat fractions based on GC-IMS data. Volatile component fingerprints of different horse fat fractions, the red indicating higher content and blue representing lower content (**a**). The quantity (**b**) and percentage (**c**) of VOCs.

HS/GC-IMS is a rapid analytical technique that combines gas chromatographic separation with ion mobility detection for volatile compounds, offering high sensitivity, fast response, simple sample preparation, and intuitive fingerprint visualization [29]. The aroma profiles of refined horse fat and the fractions obtained by dry fractionation at different temperatures were analyzed by HS-GC-IMS. As shown in Figure 2, a total of 55 compounds were identified and classified into seven groups, including alkenes, aldehyde, alcohols, ketones, esters, nitrogen-containing compounds, and others.

Among these classes, aldehydes were the most abundant group, with 11 compounds accounting for 20.00% of the total. Alkenes ranked second, with 9 compounds accounting for 16.36%. Esters and other compounds each included 8 compounds, accounting for 14.55%. Alcohols comprised 7 compounds and contributed 12.73%, while ketones and nitrogen-containing compounds each included 6 compounds, accounting for 10.91%.

The volatile profiles varied significantly among the samples. Refined horse fat and L5 were shared similar dominant compounds, mainly enriched in alcohols and aldehydes, such as 1-butanol, methyl acetate, 2-methyl, and heptanal. S25 and S5 contained higher levels of ketones, sulfur-containing compounds, and nitrogen-containing compounds, including 2-pentanone and dipropyl sulfide. S15 was dominated by ketones, sulfides, and esters, represented by 2-propanone, dimethyl sulfide, and n-propyl acetate. Overall, dry fractionation altered the distribution of volatile compounds between the solid and liquid phases, resulting in clear differences in flavor profiles among the fractions.

Aldehydes were associated with fatty, green, and grassy notes, ketones contributed buttery and creamy aromas, and esters were linked to fruity notes [30]. Sulfur-containing and nitrogen-containing compounds, which have low odor thresholds, may impart meaty, roasted, onion-like, and pungent odors [10]. Their higher levels in S25 and S15 suggested greater retention in the solid phase at 25 and 15 °C. This may be because the solid fractions retained more hydrophobic and less volatile compounds, whereas the liquid fraction contained more volatile components [11,31].

**Figure 3:**
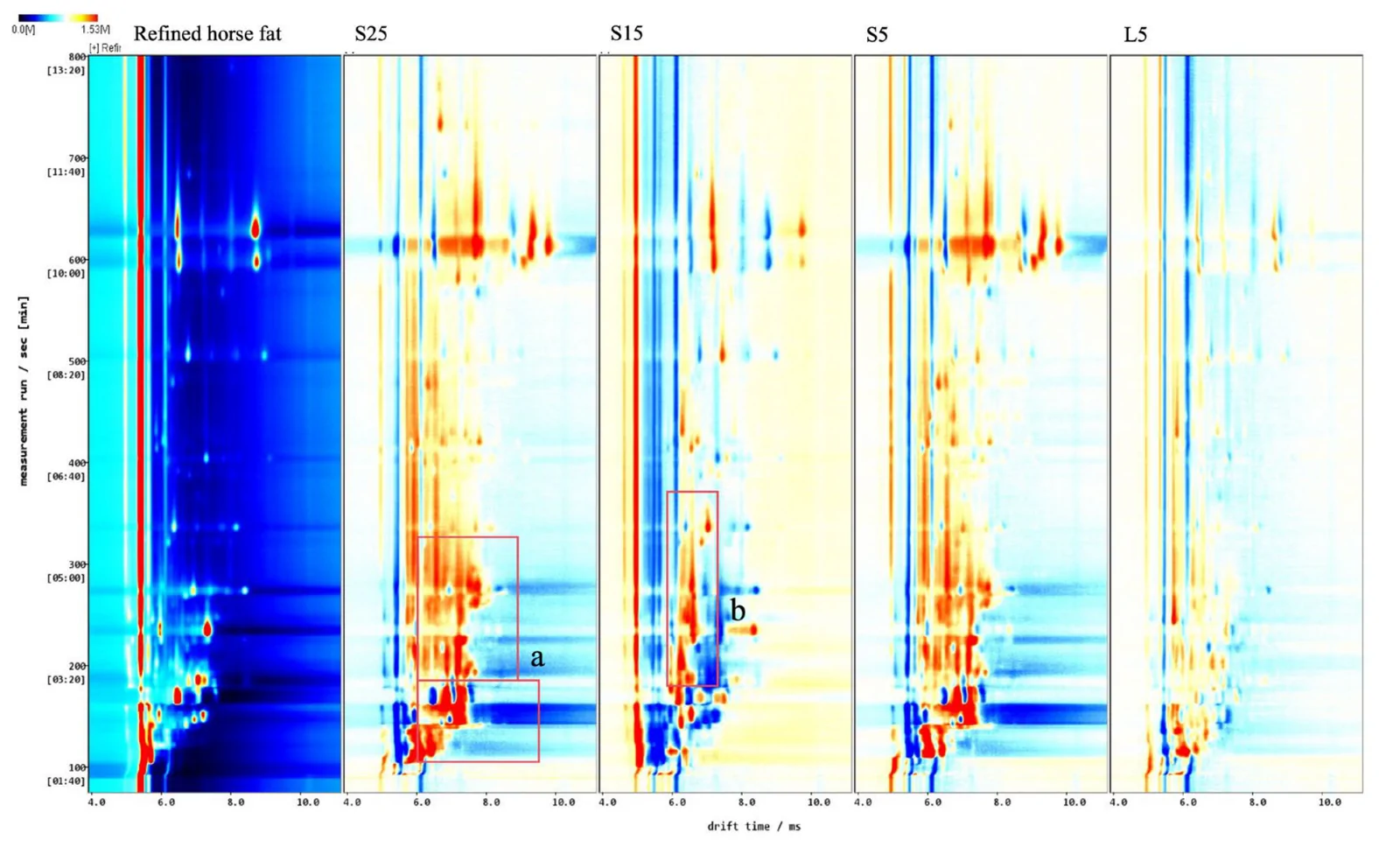
Difference view of HS-GC-IMS spectrogram. Refined horse fat was used as the control, and the spectra of the other four samples were compared against control. Blue areas indicate lower signal intensities than control, while red areas indicate higher signal intensities than control. Darker colors represent larger differences.

Difference view of HS/GC-IMS spectrogram is shown in Figure 3. Compared to refined horse fat, S25 and S5 exhibited 19 compounds with pronounced differences, mainly including aldehydes, lipids, and ketones. S15 showed 11 significantly different compounds, which were mainly ketones, lipids, and alcohols. In comparison, L5 displayed fewer differential compounds, with only five identified, namely propanal, n-propyl acetate, 2-propanone, acetic acid, and 2-methylpropanal. Accordingly, S25 and S15 showed stronger volatile compounds accumulation than L5, which was consistent with the higher E-nose responses observed for these samples. These HS-GC-IMS results were consistent with the E-nose data, which showed stronger responses in S25 and S15, particularly for sensors associated with aromatic, sulfur-containing, roasted, and fruity odorants. Overall, dry fractionation altered the distribution of volatile compounds and modified the sensory profile of horse fat.

### 3.3. Qualitative Analysis of Volatile Compounds by SPME/GC-MS

**Figure 4:**
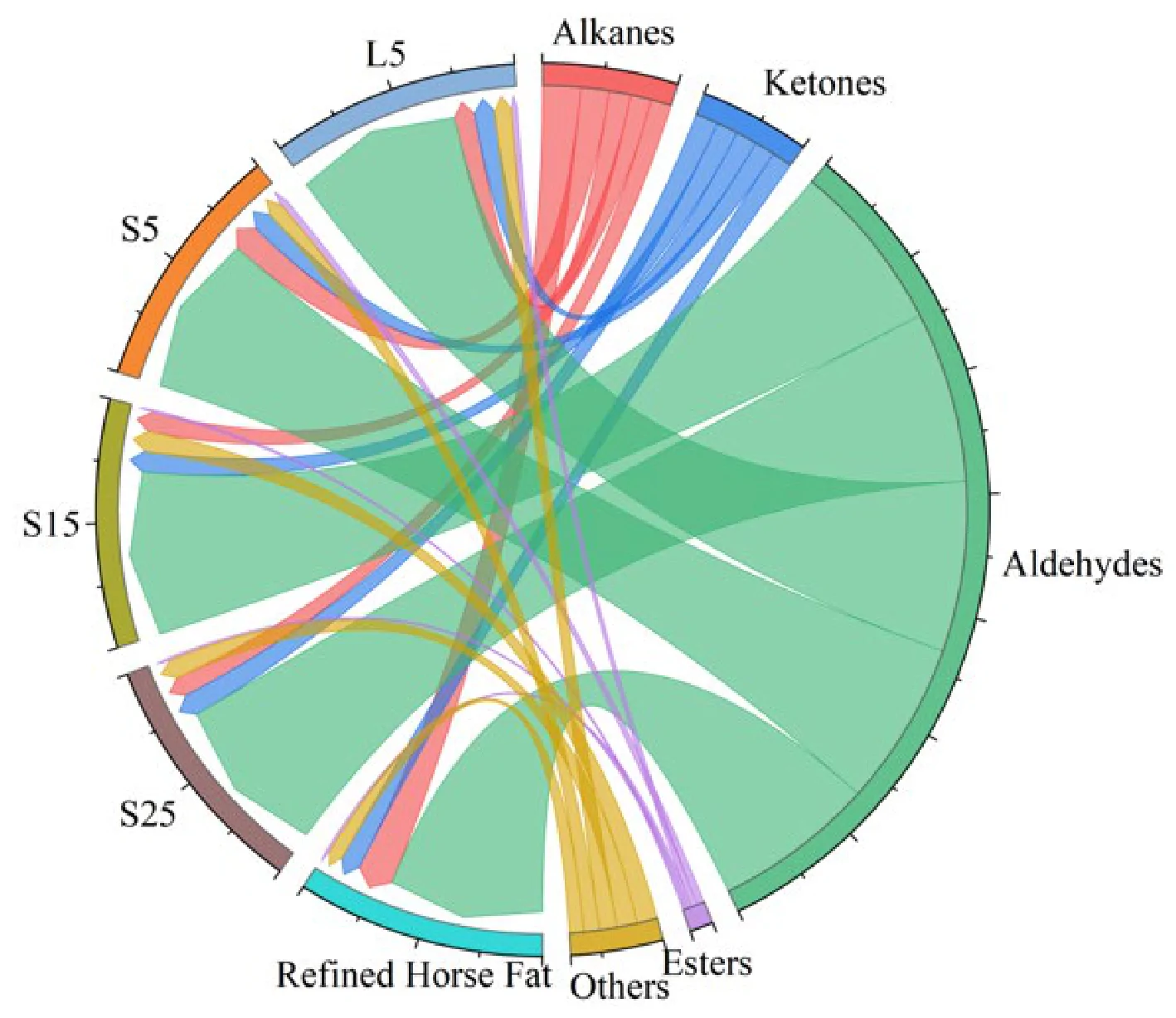
Types and relative percentages of volatile compounds in horse fat fractions by Circos plot. Refined horse fat samples after crystallization at 25, 15, and 5 °C, including liquid fractions (L5) and solid fractions (S25, S15, S5).

To have a better understanding of the flavor of the dry fractionated horse fat fractions, GC-MS was applied to analyze the volatile components. As shown in Figure 4, a total of 53 volatile compounds were identified from all refined horse fat fractions, including 20 alkanes, 8 ketones, 14 aldehydes, four esters, and seven other compounds. The specific volatiles groups and distribution in different refined horse fat fractions is shown in Figure 4. Among all samples, S5 showed the highest number of volatile species, with 42 compounds detected, followed by refined horse fat, whereas S25 and S15 exhibited the lowest complexity, each with 33 compounds. This pattern suggests that fractionation at 5 °C, especially in the solid fraction, may have retained more aroma-active compounds [10,11]. A lower crystallization temperature may have helped these minor volatile compounds enter the solid matrix [32,33]. In contrast, the reduced diversity observed in S25 and S15 may be related to the higher fractionation temperatures, which could have led to a simpler distribution of volatile components between solid and liquid phases.

**Figure 5:**
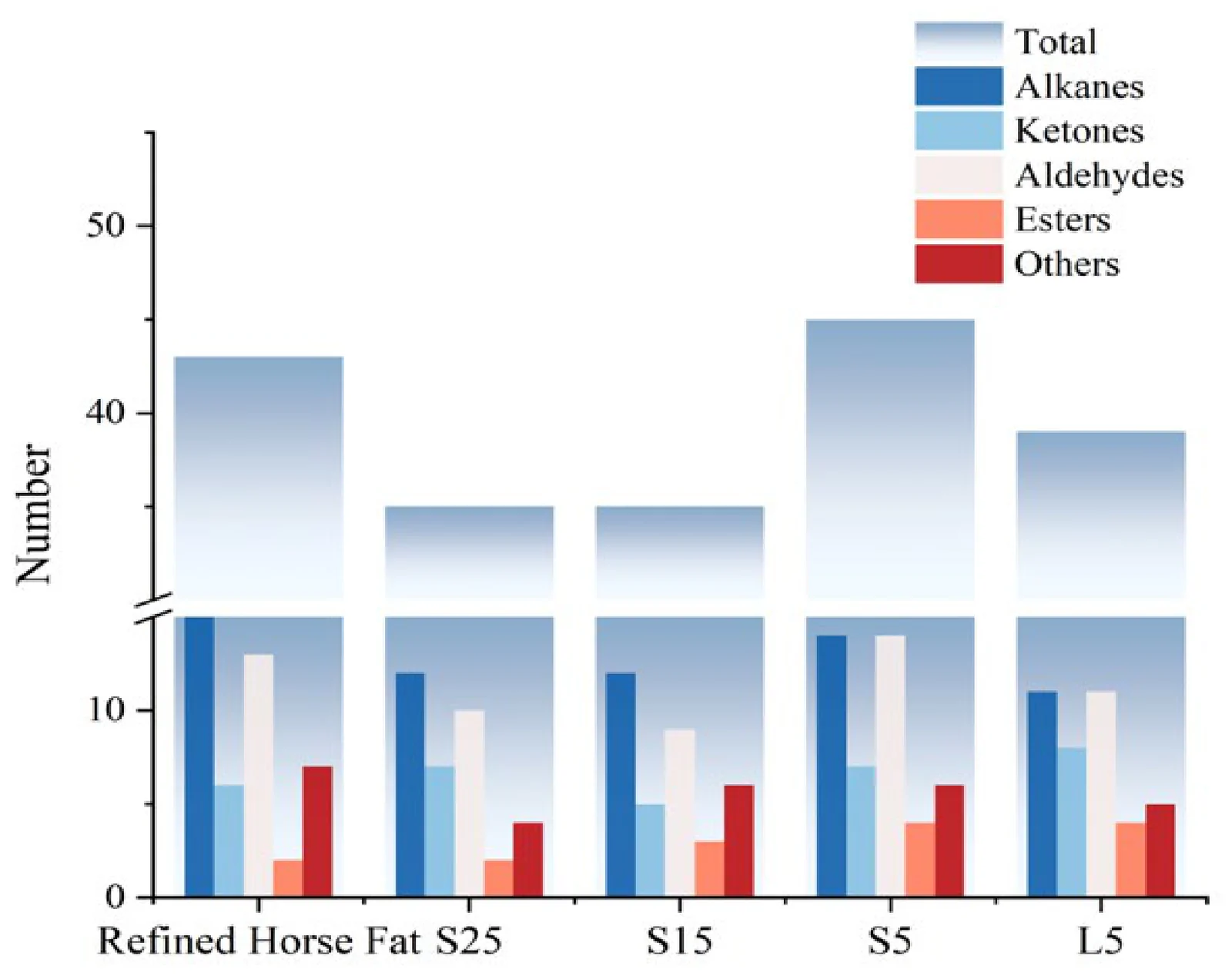
The categories and quantities of volatile compounds in each component. Refined horse fat samples after crystallization at 25, 15, and 5 °C, including liquid fractions (L5) and solid fractions (S25, S15, S5).

The stacked bar chart presented on Figure 5 shows that aldehydes were the main volatile class in all samples, accounting for 68.50% to 72.55% of the total volatiles. Alkanes were the second most abundant group, with relative contents of 8.82% to 16.19%, while esters were the least abundant. The ester content followed the order S5 > L5 > refined horse fat > S25 > S15, with values of 3.25%, 2.71%, 1.40%, 1.27%, and 1.16%, respectively.

**Figure 6:**
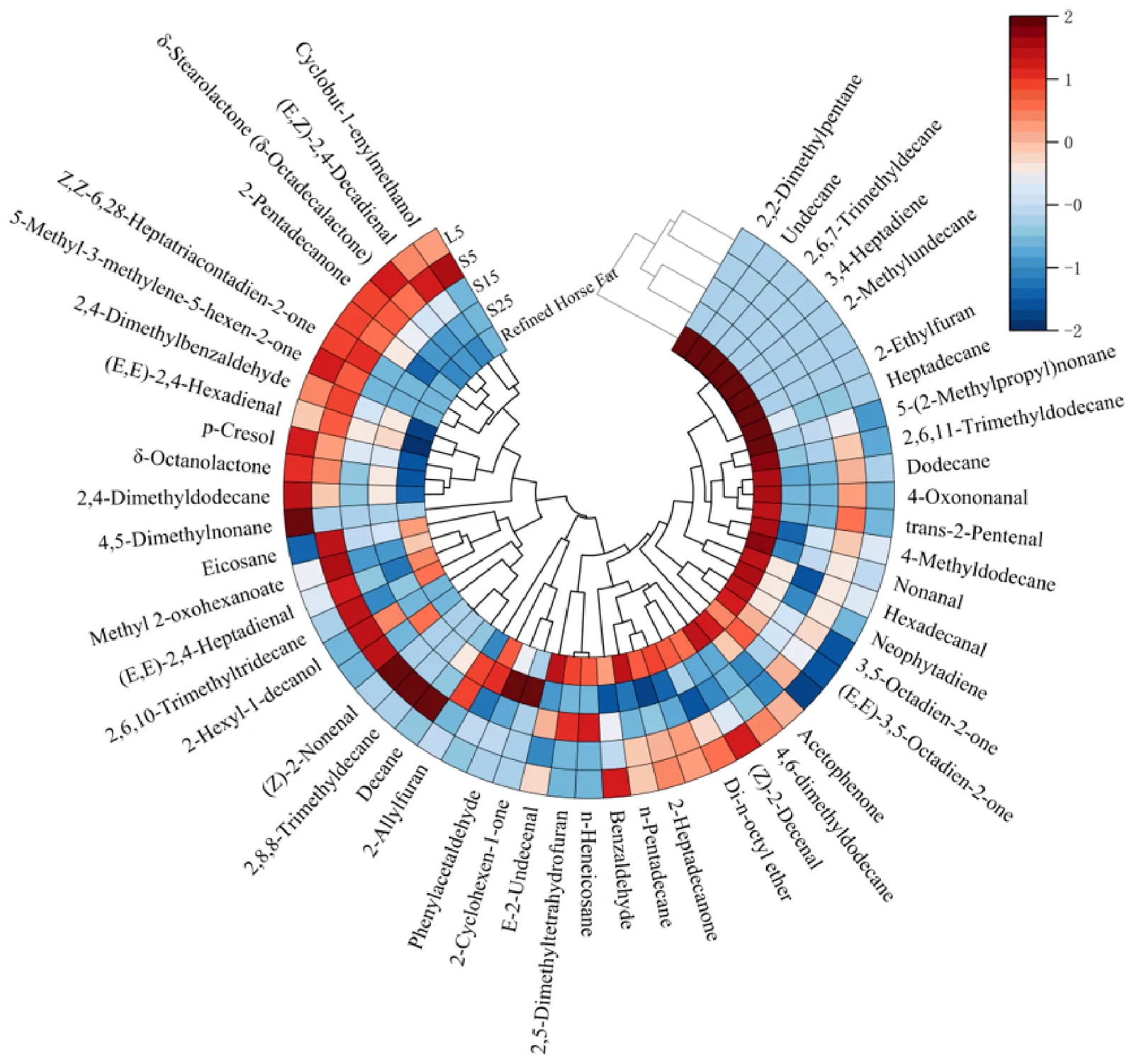
The heat map of VOCs of refined horse fat by different fractions based on GC-MS data.

The heatmap on Figure 6 shows that the volatile compounds in refined horse fat and the dry-fractionated samples showed clear differences in both amount and distribution. S5 showed the widest and strongest signal for many compounds, which suggests that fractionation at 5 °C helped retain a richer volatile profile. In contrast, S25 and S15 showed fewer strong signals and a simpler pattern, indicating that higher fractionation temperatures reduced the retention of odor-active compounds in the solid phase. L5 showed an intermediate pattern, but its signal intensity was still lower than that of S5 for many key compounds.

Similar as HS/GC-IMS, aldehydes were the main aroma compounds in all samples. They gave fatty, green, grassy, and slightly fried notes. The main aldehydes were trans-2-pentenal, (E,E)-2,4-heptadienal, nonanal, (E,Z)-2,4-decadienal, (Z)-2-decenal, and (Z)-2-nonenal, E-2-undecenal, etc. Among them, unsaturated aldehydes such as 2,4-hexadienal, 2,4-heptadienal, 2-nonenal, and 2-decenal were especially important because they are linked to strong fatty, grassy, and fried odors.

Ketones and alkanes were the second most abundant groups. Ketones mainly contributed buttery, creamy, and waxy notes, while alkanes were mostly related to weak fatty and hydrocarbon-like odors. The main ketones included 3,5-octadien-2-one, 2-pentadecanone, and 2-heptadecanone. The main alkanes included heptadecane, undecane and decane. Among these, 2-pentadecanone and 2-heptadecanone showed relatively strong signals in several samples, so they likely played an important role in the creamy and waxy aroma of horse fat.

Esters were present at lower levels, but they still added sweet, fruity, and creamy notes. Methyl 2-oxohexanoate, δ-octanolactone, δ-dodecalactone, and δ-stearolactone were the main ester-related compounds. These were more obvious in S5 than in S25 and S15, which means low-temperature fractionation helped keep more of these aroma compounds.

Other compounds also affected the aroma profile. p-Cresol gave phenolic, smoky, and animal-like notes. 2-Ethylfuran and 2-allylfuran added roasted and toasted notes. 3,4-Heptadiene and neophytadiene were linked to green and hydrocarbon-like notes. Cyclobut-1-enylmethanol and 2-hexyl-1-decanol may have added mild fatty or alcohol-like odors. In addition, sulfur- and nitrogen-related compounds, although present in small amounts, can have a strong effect on aroma because of their low odor thresholds.

Semi-quantitative analysis with 2-octanol also supported these results. The total volatile content was 16.10 μg/g in refined horse fat, 15.85 μg/g in S5, 13.30 μg/g in L5, and 11.94 μg/g and 12.25 μg/g in S25 and S15, respectively. Generally, S5 had a wider and stronger signal across many compounds than the other samples, while S25 and S15 showed fewer strong signals. This indicates that the 5 °C fraction retained a richer volatile profile [34].

Overall, the chord plot, bar chart, and heatmap showed that fractionation temperature affected both the amount and distribution of volatile compounds, with S5 showing the richest profile.

The three methods gave consistent but complementary results. E-nose showed clear differences in the overall odor pattern among samples. HS-GC-IMS identified the main marker compounds, and SPME/GC-MS provided a broader volatile profile. Together, these results show that fractionation temperature changed both the amount and the composition of volatiles in horse fat.

S25 and S15 were clearly separated from raw horse fat and the 5 °C samples by E-nose, with stronger signals for sensors related to aromatic, sulfurous, roasted, and fruity notes. This suggests that higher fractionation temperatures favored the retention of some odor-active compounds in the solid phase. HS-GC-IMS supported this result at the compound level. S25 and S15 contained more ketones, sulfur-containing compounds, and nitrogen-containing compounds, while L5 and raw horse fat were richer in aldehydes and alcohols. This indicates that lower temperatures favored the transfer of more volatile and more polar compounds into the liquid phase, while higher temperatures favored the retention of sulfur-, nitrogen-, and some carbonyl-related compounds in the solid phase. SPME/GC-MS gave a more complete view of the volatile profile. Aldehydes were the main compounds in all samples, but S5 had the highest number of volatile types and the highest total content, especially unsaturated aldehydes, ketones, and some esters. This shows that low-temperature fractionation helped retain a richer set of flavor compounds, while higher temperatures simplified the volatile profile and changed the balance between solid and liquid phases.

Overall, these results show that dry fractionation can reshape the flavor profile of horse fat. Lower temperatures helped preserve a richer and more complex aroma, while higher temperatures led to the relative enrichment of some strong odor compounds in the solid fraction.

## 4. Conclusions

Dry fractionation temperature significantly affected the volatile flavor characteristics of refined horse fat. E-nose analysis showed clear differences in the overall odor profiles of the fractions. HS-GC-IMS identified total 55 volatile compounds and indicated that S25 and S15 were enriched in ketones, sulfur-containing compounds, and nitrogen-containing compounds. HS-SPME-GC-MS further confirmed that aldehydes were the dominant volatile class in all samples, while S5 retained the richest volatile profile. These findings indicate that fractionation temperature regulates the distribution of aroma-active compounds between solid and liquid phases, thereby influencing horse fat flavor. Thus, dry fractionation offers a practical strategy for flavor control and provides guidance for the development of horse fat products with improved sensory quality.

## Data Availability

The original contributions presented in this study are included in the article. Further inquiries can be directed to the corresponding authors.
